# Varicella-Zoster Virus–Induced Neurologic Disease After COVID-19 Vaccination: A Multicenter Observational Cohort Study

**DOI:** 10.1093/ofid/ofae287

**Published:** 2024-05-24

**Authors:** Meital Elbaz, Tomer Hoffman, Dafna Yahav, Sarah Dovrat, Nesrin Ghanem-Zoubi, Alaa Atamna, Daniel Grupel, Sharon Reisfeld, Mirit Hershman-Sarafov, Pnina Ciobotaro, Ronza Najjar-Debbiny, Tal Brosh-Nissimov, Bibiana Chazan, Orit Yossepowitch, Yonit Wiener-Well, Ora Halutz, Shelley Reich, Ronen Ben-Ami, Yael Paran

**Affiliations:** Infectious Disease Unit, Tel Aviv Sourasky Medical Center, Tel Aviv, Israel; Sackler Faculty of Medicine, Tel Aviv University, Tel Aviv, Israel; Infectious Diseases Unit, Sheba Medical Center, Ramat Gan, Israel; Sackler Faculty of Medicine, Tel Aviv University, Tel Aviv, Israel; Infectious Diseases Unit, Sheba Medical Center, Ramat Gan, Israel; Central Virology Laboratory, Public Health Services, Ministry of Health, Sheba Medical Center, Tel-Hashomer, Israel; Infectious Diseases Institute, Rambam Health Care Campus, Haifa, Israel; The Ruth and Bruce Rappaport Faculty of Medicine, Technion, Haifa, Israel; Infectious Disease Unit, Rabin Medical Center, Beilinson Hospital, Petah-Tikva, Israel; Department of Clinical Microbiology and Infectious Diseases, Hadassah Medical Center, Jerusalem, Israel; Faculty of Medicine, Hebrew University of Jerusalem, Jerusalem, Israel; The Ruth and Bruce Rappaport Faculty of Medicine, Technion, Haifa, Israel; Infectious Diseases Unit, Hillel Yaffe Medical Center, Hadera, Israel; The Ruth and Bruce Rappaport Faculty of Medicine, Technion, Haifa, Israel; Infectious Diseases Unit, Bnai Zion Medical Center, Haifa, Israel; Infectious Diseases Unit, Kaplan Medical Center, Rehovot, Israel; The Ruth and Bruce Rappaport Faculty of Medicine, Technion, Haifa, Israel; Infection Control and Prevention Unit, Lady Davis Carmel Medical Center, Haifa, Israel; Infectious Diseases Unit, Assuta Ashdod University Hospital, Ashdod, Israel; Faculty of Health Science, Ben Gurion University of the Negev, Beer Sheva, Israel; The Ruth and Bruce Rappaport Faculty of Medicine, Technion, Haifa, Israel; Infectious Disease Unit, Emek Medical Center, Afula, Israel; Infectious Disease Unit, Edith Wolfson Medical Center, Holon, Tel Aviv, Israel; Faculty of Medicine, Hebrew University of Jerusalem, Jerusalem, Israel; Infectious Disease Unit, Shaare Zedek Medical Center, Jerusalem, Israel; Clinical Microbiology Laboratory, Tel Aviv Sourasky Medical Center, Tel- Aviv, Israel; Infectious Disease Unit, Tel Aviv Sourasky Medical Center, Tel Aviv, Israel; Infectious Disease Unit, Tel Aviv Sourasky Medical Center, Tel Aviv, Israel; Sackler Faculty of Medicine, Tel Aviv University, Tel Aviv, Israel; Infectious Disease Unit, Tel Aviv Sourasky Medical Center, Tel Aviv, Israel; Sackler Faculty of Medicine, Tel Aviv University, Tel Aviv, Israel

**Keywords:** COVID-19, SARS-CoV-2, vaccine safety, varicella zoster virus, zoster neurological disease

## Abstract

**Background:**

Early reports described an increased risk of herpes zoster following receipt of mRNA-based COVID-19 vaccines. The objective was to assess whether COVID-19 vaccine is associated with varicella-zoster virus–induced neurologic disease (VZV-ND).

**Methods:**

This multicenter retrospective case-control study with a test-negative design was conducted at 12 hospitals in Israel. We included all patients admitted with VZV-ND between January 2020 and December 2021 and matched controls with a negative polymerase chain reaction result for VZV in cerebrospinal fluid.

**Results:**

We identified 188 patients meeting the case definition of VZV-ND who were admitted during the study period. Cases were matched with 376 controls. There was no significant variation in the incidence of VZV-ND between 1 year preceding and 1 year following the deployment of BNT162b2 in Israel. Analysis of persons who had received at least 1 dose of COVID-19 vaccine (n = 259) showed similar proportions of VZV-ND and non–VZV-ND in 4 intervals (30, 42, 50, 60 days) following the last vaccine dose. The median time from the last vaccine dose to hospitalization with a neurologic syndrome was 53 days (IQR, 25–128) and 82 days (IQR, 36–132) for VZV-ND and non–VZV-ND, respectively, not reaching statistical significance (*P* = .056). The rate of VZV-ND in vaccinated patients was no different from the rate in the unvaccinated group (30.9% vs 35.4%, *P* = .2).

**Conclusions:**

We did not find an association between COVID-19 vaccine and VZV-ND. Since COVID-19 vaccine is now recommended yearly, every fall and winter, establishing the safety of the vaccine is of great importance.

The global effort to stop the COVID-19 pandemic generated one of the largest mass vaccination campaigns in world history. Soon after the introduction of the BNT162b2 mRNA vaccine, reports emerged of a possible association between vaccination and varicella-zoster virus (VZV) reactivation. A systematic review [1] of 12 articles following different COVID-19 vaccines reported on 91 patients with herpes zoster (HZ), with the majority being >60 years of age and more than a fifth with an autoimmune disorder and/or immunosuppression. On average, symptoms developed 5.8 days postvaccination, irrespective of dose. Barda et al [2] reported adverse events up to 42 days after vaccination in a cohort of >880 000 BNT162b2 recipients as compared with a similar number of unvaccinated controls and found an increased risk of HZ (risk ratio, 1.43; 95% CI, 1.20–1.73). Hertel et al also found a higher incidence of HZ in the 60 days after COVID-19 vaccination [3], although other studies did not confirm this association [4, 5]. Previous reports speculated that the association between COVID-19 immunization and HZ might be due to a vaccine-induced immunomodulatory effect, causing a temporary failure of VZV-specific T-cell response and leading to VZV reactivation [6–8].

VZV is a known human neurotropic herpes virus. After primary infection, VZV becomes latent in ganglionic neurons along the entire neuraxis. With a decline in VZV-specific cell-mediated immunity, latent VZV in dorsal root ganglia may reactivate, migrating anterograde to the skin to cause zoster, which is often complicated by postherpetic neuralgia. VZV can also migrate retrograde to the central nervous system (CNS), where it produces VZV-induced neurologic disease (VZV-ND), which can manifest as meningoencephalitis, myelitis, polyradiculitis, and stroke [9, 10].

Soon after the association between COVID-19 immunization and HZ was reported, the question was raised whether the vaccine is associated VZV-induced neurologic disease. Several case reports [11–13] described neurologic complications of VZV after vaccination with a COVID-19 vaccine in patients who were immunocompetent, within 5 to 21 days of vaccination. These reports described 4 patients without known immunosuppression, 3 of whom were <50 years of age, who developed VZV meningitis after COVID-19 vaccine. Two of the 4 patients did not receive prior zoster vaccination (information is missing for the other 2 patients). Despite these reports, causality remains uncertain, and studies [8] examining the association were small and unpowered to detect a significant association between the vaccine and VZV-ND.

The rapid deployment of BNT162b2 to the Israeli population between December 2020 and June 2021 offers an opportunity to assess the association of vaccination with VZV-ND. Here, we report the results of a multicenter case-control study aimed at exploring this association.

## METHODS

### Study Design and Population

This multicenter retrospective case-control study with a test-negative design was conducted in 12 hospitals in Israel, including 6 of the 7 tertiary medical centers in the country. We collected data on hospitalized patients at least 18 years of age admitted to participating sites with proven VZV-ND between 1 January 2020 and 31 December 2021.

Proven VZV-ND was defined as a positive polymerase chain reaction (PCR) test result for VZV from cerebrospinal fluid (CSF), abnormal CSF analysis (pleocytosis and/or elevated protein), and a compatible clinical syndrome: meningitis, encephalitis, meningoencephalitis, radiculitis/polyradiculitis, cerebellitis, myelitis, and CNS vasculopathy, with or without HZ (sine herpete). The control group comprised patients with 1 or more of the aforementioned neurologic syndromes, pathologic CSF analysis, negative PCR result for VZV, and no evidence of bacterial or fungal CNS infection. The control group consisted only of patients who underwent CSF analysis in the first 10 days of hospitalization. Cases were matched to controls by sex and age (±10 years) at a ratio of 1:2.

Exclusion criteria included HZ ophthalmicus and Ramsay Hunt syndrome (HZ oticus) as the sole neurologic complication of VZV. Although both these syndromes are neurologic complications of VZV, we decided to exclude them since the diagnosis is mostly clinical and in the majority of cases lumbar puncture and CSF examinations are not performed; as such, the search of positive PCR results for VZV in CSF would have missed many of these patients. We also excluded patients admitted with bacterial or fungal CNS infection, specifically at least 1 of the following: positive bacterial cultures from CSF, blood culture positive for bacteria (eg, *Listeria monocytogenes*, *Streptococcus pneumoniae*), positive PCR result for bacteria or fungi, or positive cryptococcal antigen from CSF.

To examine a possible association between COVID-19 immunization and VZV-ND, we calculated the odds of receiving at least 1 dose of vaccine in VZV-ND and non–VZV-ND cases at different time intervals prior to CSF testing (30, 42, 50, and 60 days). A secondary outcome in this analysis was the median time from vaccination to hospitalization with VZV-ND vs non–VZV-ND. To reject the null hypothesis of no association between COVID-19 vaccination and VZV-ND, it was expected that median time from vaccine to VZV-ND would be significantly different from the median time from vaccine to non–VZV-ND.

The study was reviewed and approved by the ethics committee of each participating site (approval for the principal site, 0825-21TLV). Requirement for informed consent was waived considering the retrospective observational nature of the study.

### Data Collection

Data were retrieved from the electronic medical record system and laboratory computerized database of each center. Collected variables were as follows: demographic data; comorbidities quantified by the Charlson comorbidity score [14]; immunosuppression, including chemotherapy, HIV/AIDS, solid organ transplantation, hematopoietic stem cell transplantation, glucocorticoid therapy (>20-mg/d prednisone or equivalent for ≥3 weeks), neutropenia (absolute neutrophil count <500/µL in the 14 days prior to symptom onset), asplenia, and treatment with monoclonal antibodies or other immunosuppressive drugs; and prior COVID-19 infection and COVID-19 vaccination status (ie, number of doses prior to hospitalization, last dose date, and type of vaccine). Variables associated with index hospitalization included time from last vaccine dose to hospitalization, type of clinical neurologic syndrome, and laboratory parameters of CSF analysis.

### Statistical Analysis

Patient and disease variables were described within each patient cohort (VZV-ND and control) by number and percentage for categorical variables and median and IQR for continuous variables. Between-group differences were assessed with a Fisher exact test for categorical variables and a Student *t* test or Wilcoxon rank sum test for normally and nonnormally distributed continuous variables, respectively.

A temporal association between the COVID-19 vaccination campaign and VZV-ND was assessed by analyzing the epidemic curve of VZV-ND. A 7-day moving average of new VZV-ND cases was calculated, and interrupted time series analysis was performed to detect variation in incidence following rollout of the BNT162b2 vaccine in December 2020.

The association between VZV-ND and patient demographics, comorbidities, and clinical and laboratory variables was assessed by binomial regression. Variables that were associated with VZV-ND status (*P* < .1) were further assessed by constructing a multivariable binomial regression model. Receipt of COVID-19 within 30 days prior to onset of neurologic disease, coded as a categorical variable, was forced into the model. Model goodness of fit was tested with the Hosmer-Lemeshow statistic.

## RESULTS

An overall 188 patients meeting the case definition of VZV-ND were identified by screening the medical record databases of 12 participating centers from 1 January 2020 through 31 December 2021. Case patients were matched with 376 controls (1:2) according to criteria detailed in the Methods section. In the control group, 24.5% of patients (92/376) had an alternative infectious etiology.

Patient characteristics are presented in Table 1. The median age was 55.7 years (IQR, 35–73.5) and 306 (54%) were male. Ninety patients (16%) were immunosuppressed and 33 (5.8%) had autoimmune disease, with no significant differences between groups. Twenty-three patients (4%) had previous COVID-19.

**Table 1.** Characteristics of Patients With VZV-ND and Non–VZV-ND

	VZV-ND (n = 188)	Non–VZV-ND (n = 376)	Total (n = 564)	*P* Value
Age, y	55.8 (35–73.9)	55.7 (33.8–73.1)	55.7 (35–73.5)	.5
Male	102 (54)	204 (54)	306 (54)	>.99
Charlson Comorbidity Index	1 (0–4.5)	2 (0–5)	2 (0–5)	.9
Immunosuppression^a^	24 (12.8)	66 (17.5)	90 (16)	.17
Autoimmune disease	12 (6.4)	21 (5.5)	33 (5.8)	.7
Previous COVID-19	7 (3.7)	16 (4.2)	23 (4)	.8
Vaccination doses	80 (42.5)	179 (47.6)	259 (45.9)	.2
1	14 (17.5)	24 (13.4)	38 (14.7)	.4
2	45 (56.2)	105 (58.6)	150 (57.9)	.7
3	21 (26.3)	49 (27.4)	70 (27)	.8
Neurologic disease				
Meningitis	112 (59.6)	152 (40.4)	254 (45)	<.01
Encephalitis/meningoencephalitis	60 (31.9)	160 (42.6)	220 (39)	.002
Myelitis/encephalomyelitis	5 (2.6)	17 (4.5)	22 (3.9)	.3
Radiculitis	5 (2.6)	1 (0.25)	7 (1.2)	.004
Other neurologic syndrome	6 (3.2)	46 (12.2)	25 (4.4)	
CSF analysis				
White blood cells, cells/mm^3^	158 (38–360)	22 (7–100)	…	<.001
PMNs, %	1 (0–4)	11.9 (1–36)	…	<.001
Mononuclear cells, %	99 (96–100)	86 (56–99)	…	<.001
Protein, mg/dL	96.1 (66.2–141.2)	67 (47.4–103.3)	…	<.01
Hypoglycorrhachia^b^	27 (14.4)	27 (7.2)	…	.009
Microbiology				
HSV-1	…	10 (2.7)	…	…
HSV-2	…	3 (0.8)	…	…
Enterovirus	…	35 (9.3)	…	…
Epstein-Barr virus	…	2 (0.5)	…	…
Cytomegalovirus	…	0	…	…
HHV-6	…	3 (0.8)	…	…
West Nile virus	…	39 (10.4)	…	…
No pathogen identified	…	284 (75.5)	…	…

Categorical variables are presented as No. (%) and continuous variables as median (IQR).

Abbreviations: CSF, cerebrospinal fluid; HHV, human herpes virus; HSV, herpes simplex virus; PMN, polymorphonuclear leukocyte; VZV-ND, varicella-zoster virus–induced neurologic disease.

^a^Immunosuppression: chemotherapy, HIV/AIDS, solid organ transplantation, hematopoietic stem cell transplantation, glucocorticoid therapy (>20-mg/d prednisone or equivalent for ≥3 weeks), neutropenia (absolute neutrophil count <500/µL in the 14 days prior to a positive polymerase chain reaction test result for VZV), asplenia, and treatment with monoclonal antibodies or other immunosuppressive drugs.

^b^Hypoglycorrhachia: CSF glucose level <45 mg/dL or a CSF:serum glucose ratio ≤0.4.

Most patients with VZV-ND had meningitis (112/188, 59.6%), followed by encephalitis/meningoencephalitis (31.9%) and radiculitis (2.6%; Table 1). Patients with VZV-ND had pronounced mononuclear pleocytosis (median, 99% mononuclear cells), with 14.4% of patients presenting with hypoglycorrhachia.

Rash was present in 114 patients (60.6%). In 54 patients, the rash involved the head and neck; 30 had thoracic rash, 13 lumbar, and 11 sacral; and 6 patients had disseminated rash.

Almost all patients with VZV-ND (181/188, 96.3%) received antiviral therapy: 175 were treated with acyclovir and 6 with valacyclovir. The other 7 patients had meningitis, with information regarding treatment missing for 5 of them; the other 2 improved spontaneously and were discharged before PCR results.

The median length of hospitalization in the VZV-ND group was 10 days (IQR, 6–14). Neurologic sequelae were present at discharge in 43 patients (22%): 25 (13.3%) had focal neurologic deficits, 9 (4.8%) had postherpetic neuralgia, and 9 (4.8%) had cognitive impairment. In-hospital mortality in the VZV-ND group was 5.9% (11/188).

### Association Between COVID-19 Vaccination and VZV-ND

**Figure 1.**
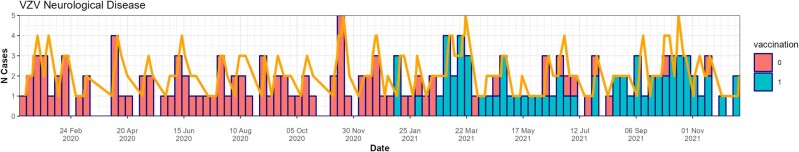
Epidemiologic curve. VZV neurologic cases in different periods during 2020 to 2021 in unvaccinated (red) and vaccinated (blue) patients. Line shows the weekly moving average of cases of VZV-induced neurologic disease. VZV, varicella-zoster virus.

Most patients in both groups received 2 vaccine doses prior to hospitalization (56.25% in the VZV-ND group and 58.6% in the control group, *P* = .7). Most vaccinated patients received the Pfizer BNT162b2 mRNA COVID-19 vaccine (n = 255, 98%). Figure 1 shows the epidemiologic curve and weekly incidence of hospitalizations with VZV-ND of vaccinated vs unvaccinated patients over time.

The epidemiologic curve spanning the period starting 1 year before the start of the COVID-19 immunization campaign until 1 year after that date showed no temporal clustering of VZV-ND cases suggestive of vaccine-associated events (Figure 1). The median weekly incidence of VZV-ND was 2 (IQR, 2–3) in the pre- and postvaccine periods. Interrupted time series analysis showed no significant variation in VZV-ND incidence between periods preceding and following rollout of the BNT162b2 vaccine (*F* = 1.58; 95% CI, .02–8.60; *P* = .19).

Analysis of persons who had received at least 1 dose of COVID-19 vaccine (n = 259) showed similar proportions of VZV-ND and non–VZV-ND in each of the 4 time intervals following the last vaccine dose (Table 2). The rate of VZV-ND was 30.9% (80/259) in vaccinated patients vs 35.4% (108/305) in the unvaccinated group (*P* = .2).

**Table 2.** Rate of Vaccinated Patients in Different Periods Between Last Vaccine Dose and Admission With Neurologic Disease in VZV-ND and Non–VZV-ND

	VZV-ND (n = 80)	Non–VZV-ND (n = 179)	All (n = 259)	*P* Value
Time since last COVID-19 vaccine, d				
<30	21 (12.1)	36 (9.5)	59 (10.4)	.38
<42	32 (16.8)	54 (14.2)	86 (15.1)	.45
<50	38 (20.0)	59 (15.6)	97 (17.0)	.19
<60	43 (22.6)	67 (17.7)	110 (19.3)	.17

Abbreviation: VZV-ND, varicella-zoster virus–induced neurologic disease.

Factors associated with VZV-ND on univariate analysis (Table 3) included prior corticosteroid treatment (odds ratio [OR], 0.43; *P* = .06), concomitant HZ rash (OR, 531.41; *P* < .001), meningitis syndrome (OR, 2.46; *P* < .001), encephalitis syndrome (OR, 0.64; *P* = .02), radiculitis (OR, 4.86; *P* = .06), CSF leukocyte count (OR, 1.0029; *P* < .001), CSF mononuclear fraction (OR, 1.02; *P* < .001), polymorphonuclear leukocyte percentage in CSF (OR, 0.96; *P* < .001), CSF protein (OR, 1.002; *P* = .03), CSF glucose (OR, 0.98; < .001).

**Table 3.** Univariant Analysis of Factors Associated With VZV-ND

Factor	Odds Ratio (95% CI)	*P* Value
COVID-19 vaccine 30 d prior to hospitalization	1.43 (.82–2.48)	.2
Vaccine dose number	1.11 (.89–1.38)	.4
Male sex	0.99 (.69–1.41)	.9
Age	1.0028 (.9945–1.0113)	.5
Comorbidity		
Ischemic heart disease	0.84 (.41–1.75)	.6
Congestive heart failure	1.12 (.57–2.22)	.7
Peripheral vascular disease	0.5 (.14–1.78)	.3
Cerebrovascular accident/transient ischemic attack	1.06 (.57–1.97)	.8
Dementia	1.5 (.72–3.15)	.3
Chronic pulmonary disease	0.77 (.32–1.89)	.5
Connective tissue disease	1.67 (.68–4.13)	.3
Diabetes mellitus	1.06 (.67–1.66)	.8
Leukemia	1.74 (.58–5.28)	.3
Lymphoma	1.21 (.43–3.4)	.7
Solid tumor	0.84 (.39–1.8)	.6
Chronic kidney disease	0.81 (.36–1.83)	.6
Liver disease	1.01 (.09–11.26)	.9
Neutropenia,^a^ ANC <500/µL	0.4 (.05–3.44)	.4
Asplenia	0.978	.9
Corticosteroid therapy^b^	0.43 (.17–1.05)	.06
Current hospitalization with VZV-ND		
Concomitant herpes zoster rash	531.41 (73.04–3866.18)	<.001
VZV		
Meningitis	2.46 (1.63–3.73)	<.001
Encephalitis	0.64 (.44–.94)	.02
Myelitis	0.54 (.19–.48)	.2
Radiculitis	4.86 (.93–25.31)	.06
CSF		
Pleocytosis	1.0029 (1.002–1.0039)	<.001
PMNs, %	0.96 (.95–.98)	<.001
Mononuclear cells, %	1.02 (1.01–1.03)	<.001
Protein	1.002 (1.0001–1.0038)	.03
Glucose	0.98 (.97–.99)	<.001

Abbreviations: ANC, absolute neutrophil count; CSF, cerebrovascular fluid; PMN, polymorphonuclear leukocyte; VZV-ND, varicella-zoster virus–induced neurologic disease.

^a^Neutropenia: ANC <500/µL in the 14 days prior to a positive polymerase chain reaction test result for VZV.

^b^Glucocorticoid therapy: >20-mg/d prednisone or equivalent for ≥3 weeks.

The following were not associated with VZV-ND on univariant analysis: age, sex, vaccine 30 days prior to index hospitalization, number of vaccine doses, comorbidities (ischemic heart disease, cerebrovascular accident, chronic pulmonary disease, congestive heart failure, peripheral vascular disease, diabetes mellitus, connective tissue disease, chronic kidney disease, chronic liver disease, hemato-oncologic disease), dementia, and neutropenia. HZ rash, CSF pleocytosis, monocytosis in CSF, and low glucose in CSF remained significant predictors of VZV-ND on multivariant analysis (Table 4).

**Table 4.** Multivariant Analysis of Patients With Varicella-Zoster Virus–Induced Neurologic Disease

	Odds Ratio (95% CI)	*P* Value
Herpes zoster rash	424.8 (79.4–8090.4)	<.001
CSF		
White blood cell count	1.002 (1.00098–1.0032)	<.001
Monocyte percentage	1.020 (1.0099–1.033)	<.001
CSF:serum glucose ratio	0.040 (.0024–.58)	.002
COVID-19 vaccination	1.56 (.57–4.020)	.35

Abbreviation: CSF, cerebrospinal fluid.

**Figure 2.**
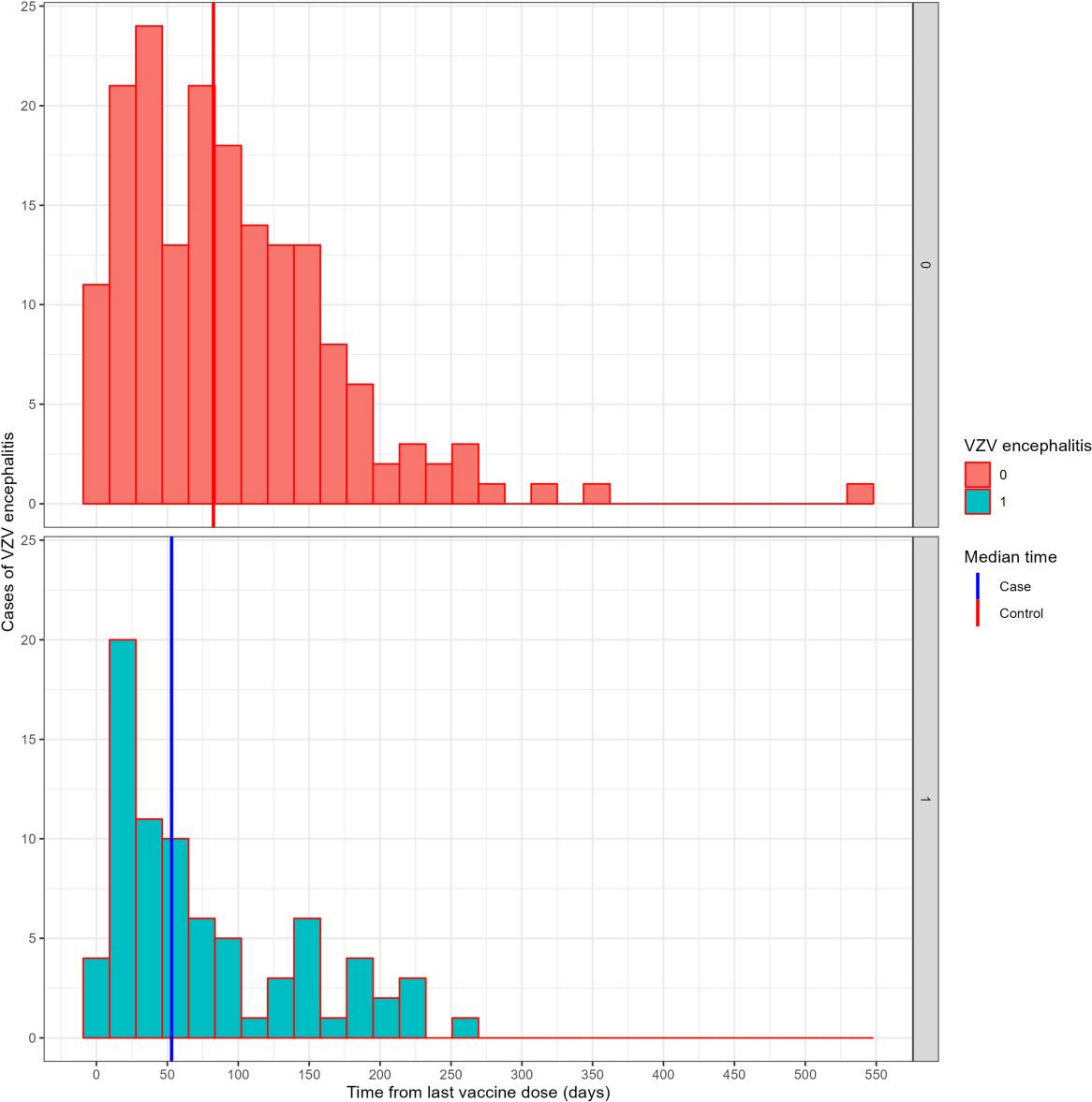
Time from last COVID-19 vaccine dose to hospitalization with a neurologic syndrome in patients with (green) and without (red) VZV-induced neurologic disease. VZV, varicella-zoster virus.

The median time from the last vaccine dose to hospitalization with neurologic syndrome was 53 days (IQR, 25–128) and 82 days (IQR, 36–132) for VZV-ND and non–VZV-ND, respectively (Figure 2), not reaching statistical significance (*P* = .056).

## DISCUSSION

In the present study, we retrospectively evaluated all patients who were admitted to 12 medical centers in Israel with a diagnosis of VZV-ND during the first year of the COVID-19 vaccination campaign (January–December 2021) and in the same period in the previous year, and we compared them with patients with non–VZV-ND to examine a possible association between vaccination with a COVID-19 vaccine and VZV-ND.

An overall 188 patients meeting the case definition of VZV-ND were identified during the study period, with VZV meningitis being the most common CNS syndrome, corresponding with other reports describing VZV as one of leading causes of viral meningitis with enteroviruses and herpes simplex virus [15]. The median CSF white blood cell count was 158 (IQR, 38–360) with hypoglycorrhachia present in 14.4% of patients, corresponding with previous reports that the CSF profile in VZV-ND may sometimes mimic pyogenic infection [16, 17], suggesting that CNS infection with a high cell count and low glucose level does not rule out viral infection, such as VZV-ND.

There was no significant difference in the proportion of vaccinated patients in the VZV-ND group vs the non–VZV-ND group for all 4 periods before admission. The median interval from the last COVID-19 vaccine dose was marginally shorter for VZV-ND vs non–VZV-ND, bordering on statistical significance. The vaccine was not a predictor of VZV-ND and remained so in multivariant analysis. In a second analysis, there was no significant difference between the rate of VZV-ND in the vaccinated group and the rate in the unvaccinated group; there was actually a trend toward less VZV-ND in the vaccinated group.

The COVID-19 immunization campaign in Israel was characterized by rapid uptake in the target population. By 10 April 2021, >10 million doses of the vaccine had been administered, and >70% of Israelis aged ≥16 years had received 2 doses [18]. The rapid vaccine rollout provides an optimal condition to detect signals of vaccine-associated adverse events. Despite this, the epidemiologic curve of VZV-ND cases in 2020 to 2021 does not show any obvious increase or clustering in VZV-ND cases in the period after the onset of the vaccine campaign.

Since COVID-19 has now become an endemic disease worldwide and vaccine is now recommended yearly, every fall and winter, establishing the safety of the vaccine is of great importance. To our knowledge, only 1 study has addressed the issue of a possible association between COVID-19 vaccination and VZV-ND. Abu-Rumeileh et al [8] investigated this connection between VZV-ND and COVID-19 immunization in a single-center retrospective study in Germany. They used a case-control design and compared the number of patients with VZV-ND in a period after the introduction of the COVID-19 vaccine in 2021 against the same months in the previous 2 years. Three of 9 patients in 2021 had received their COVID-19 vaccine 6 weeks before the VZV-ND presentation, and there was a mild increase of total VZV-ND cases that year; however, the study was too small to assess the possible association between COVID-19 vaccination and VZV-ND. The authors concluded that larger studies should be done. The present multicenter nationwide study, done on a much larger cohort of patients, did not reveal a significant association.

Our study has some limitations. One is that the timing between onset of symptoms and CSF sampling was not documented, so there is a small chance that the control group included aa few patients with VZV-ND and negative CSF PCR result. We believe that most patients hospitalized with acute neurologic symptoms and suspected CNS infection will undergo lumbar function soon after hospital arrival. Moreover, in 95% of cases, viral DNA can be detected during the first week of the clinical disease [19], and in our study the control group consisted only of patients who underwent CSF analysis in the first 10 days of hospitalization; thus, we assume that even if some cases of VZV infection were missed, it would be of minor significance.

As mentioned earlier, there was a trend toward a shorter period between vaccination and hospitalization in the VZV- ND group as compared with the control group. Although this difference was not statistically significant, it is possible that because VZV-ND is a rare disease, with an estimated annual incidence of 1.02 per 100 000 [20], this study may have been underpowered to detect such difference. Yet, this was a large multicenter study that collected cases over 2 years in which COVID-19 vaccination was widely distributed; as such, if an association was missed, then this potential association is extremely low.

In conclusion, in this large multicenter study based on a large cohort, we did not find an association between COVID-19 vaccine and VZV-ND. Due to the low incidence of VZV-ND, a weak association cannot be excluded and so might require a larger study.

## Notes

***Financial support*.** This research received no specific grant from any funding agency in the public, commercial, or not-for-profit sectors.
